# Effects of *Lycium barbarum* glycopeptide on renal and testicular injury induced by di(2-ethylhexyl) phthalate

**DOI:** 10.1007/s12192-022-01266-0

**Published:** 2022-04-01

**Authors:** Xianling Zhou, Zhigang Zhang, Heng Shi, Qiubo Liu, Yuling Chang, Weifeng Feng, Shiping Zhu, Shengyun Sun

**Affiliations:** 1 Department of Nephrology, The First Affiliated Hospital of Jinan University, 613 Huangpu Avenue West, Guangzhou, 510630 Guangdong China; 2 School of Traditional Chinese Medicine, Jinan University, Guangzhou, 510630 China; 3 Department of Traditional Chinese Medicine, The First Affiliated Hospital of Jinan University, Guangzhou, 510630 China

**Keywords:** Di(2-ethylhexyl) phthalate, *Lycium barbarum* glycopeptide, Kidney, Testis, Autophagy, Inflammation

## Abstract

Di(2-ethylhexyl) phthalate (DEHP) is a common environmental pollutant with renal and reproductive toxicity. *Lycium barbarum* glycopeptide (LbGp) is the main active component of *Lycium barbarum*, which can protect the kidney and promote reproduction. Autophagy and apoptosis are the regulatory mechanisms of cell adaptation to external stress. This study investigated whether DEHP and LbGp affect kidney and testis by regulating autophagy and apoptosis. DEHP induced apoptosis in human embryonic kidney-293 (HEK-293) cells and human kidney-2 (HK-2) cells, as well as glomerular enlargement, enhanced renal autophagy and inflammation, decreased testicular germ cells, and enhanced testicular autophagy. LbGp reduced apoptosis in HEK-293 cells and HK-2 cells, reduced glomerular enlargement and renal inflammation, enhanced renal autophagy, increased testicular germ cells, and alleviated testicular autophagy. These results suggested that DEHP induced inflammation to cause kidney injury, mildly enhanced renal autophagy, and also induced excessive autophagy, leading to testicular injury. LbGp reduced inflammation and appropriately enhanced autophagy to alleviate renal injury and also reduced excessive autophagy to alleviate testicular injury. Silent information regulator 1 (SIRT1)/forkhead box O3a (FoxO3a)-mediated autophagy and p38 mitogen-activated protein kinase (p38 MAPK)-mediated inflammation played important roles.

## Highlights

- Lycium barbarum glycopeptide can reduce di(2-ethylhexyl) phthalate-induced apoptosis of human embryonic kidney-293 cells and human kidney-2 cells
- Lycium barbarum glycopeptide ameliorates di(2-ethylhexyl) phthalate-induced renal injury in mice by reducing p38 MAPK-mediated inflammation and enhancing SIRT1/FoxO3a-mediated autophagy.
- Lycium barbarum glycopeptide ameliorates di(2-ethylhexyl) phthalate-induced testicular injury in mice by reducing SIRT1/FoxO3a-mediated excessive autophagy.

## Introduction

Di(2-ethylhexyl) phthalate (DEHP) is a common environmental pollutant that is present in various plastic products (Erythropel et al. 2014; Gao and Wen 2016; Kashyap and Agarwal 2018; Lü et al. 2018; Yang 2019). It can enter the body through the respiratory and digestive tracts and is toxic to multiple organs (Kashyap and Agarwal 2018; Luo et al. 2018, 2020). DEHP can induce autophagy and apoptosis in mouse testicular cells (Barakat et al. 2017; Sun et al. 2018; Wei et al. 2018; Pan et al. 2019; Balci et al. 2020; Gan et al. 2020; Zhang et al. 2020; Zhao et al. 2020; Zhu et al. 2021) and damage to human sperm DNA, leading to apoptosis (Wang et al. 2016a, 2016b, 2019b). A low dose of DEHP can properly activate autophagy to protect the testis, while a high dose of DEHP can mediate apoptosis to damage the testis (Fu et al. 2020). DEHP can induce apoptosis of mouse renal cells and human embryonic kidney-293 (HEK-293) cells (Amara et al. 2019, 2020). Autophagy and apoptosis are the regulatory mechanisms for cells to adapt to external stress and jointly maintain cell homeostasis (Elmore 2007; Maiuri et al. 2007; Mizushima et al. 2008; Song et al. 2017). The mechanism by which DEHP induces renal and testicular autophagy and apoptosis remains unclear.

According to the Chinese pharmacopoeia, *Lycii Fructus*, as a traditional Chinese medicine, can enrich the liver and kidney and treat impotence and spermatorrhea. *Lycium barbarum* glycopeptide (LbGp) is the main active component of *Lycii Fructus*, including LbGp1 (88 kDa) (Tian et al. 1995), LbGp2 (68.2 kDa) (Peng and Tian 2001), LbGp3 (92.5 kDa), LbGp4 (214.8 kDa), and LbGp5 (23.7 kDa) (Huang et al. 1998). LbGp can induce the apoptosis of cancer cells and aged T cells (Yuan et al. 2008; Gong et al. 2020), enhance the function of macrophages (Gong et al. 2018), accelerate the proliferation of mouse splenic cells and the survival of human fibroblasts (Peng et al. 2001; Zhao et al. 2005), inhibit the apoptosis of the seminiferous epithelium (Wang et al. 2002), and enhance the proliferation of mouse spleen lymphocytes (Deng et al. 2003; Du et al. 2004). It is not distinct whether LbGp is able to improve DEHP-induced damage by adjusting apoptosis and autophagy.

Silent information regulator 1 (SIRT1) is a nicotinamide adenine dinucleotide (NAD)-dependent deacetylase relevant in the management of apoptosis, autophagy, inflammation, stress, metabolism, and aging (Rahman and Islam 2011; Kong et al. 2015). SIRT1 induces the deacetylation of forkhead box O3a (FoxO3a), which enhances autophagy, fights oxidation, inhibits apoptosis (Hori et al. 2013; Wang et al. 2016c; Tia et al. 2018), and ameliorates renal injury (Brunet et al. 2004; Kume et al. 2010; Murtaza et al. 2017; Liu et al. 2018; Wang et al. 2019a; Dusabimana et al. 2020; Zhu et al. 2020; Li et al. 2021; Meng et al. 2021). Microtubule-associated protein light chain 3 (LC3) is widely used to detect autophagy and is significantly associated with autophagosomes (Mizushima and Yoshimori 2007; Menzies et al. 2012; Klionsky et al. 2016). Cleaved caspase-3 is the active form of apoptosis marker caspase-3, which degrades a variety of cellular proteins and DNA (Kamada et al. 2005; McIlwain et al. 2013; Bernard et al. 2019). Mitogen-activated protein kinase (MAPK) signaling is relevant in cell differentiation, cell proliferation, and adaptation to environmental stress (Pearson et al. 2001). p38 MAPK is a subgroup of MAPKs that can induce inflammation and fibrosis and lead to kidney damage (Zeng et al. 2020; Arab et al. 2021; Lindfors et al. 2021; Qin et al. 2021). Various stresses can cause body damage by inducing inflammation (Hussain et al. 2016; Wirtz and von Känel 2017; Atrooz and Salim 2020). Whether DEHP and LbGp affect the kidney and testis through SIRT1/FoxO3a and p38 MAPK signaling is unknown.

In this research, we analyzed the role of SIRT1/FoxO3a-mediated apoptosis and autophagy and p38 MAPK-mediated inflammation in DEHP-induced renal and testicular injury and assessed the use of LbGp in improving renal and testicular injury.

## Materials and methods

### Drugs and reagents

LbGp powder was purchased from Ningxia Tianren Goji Biotechnology Co., Ltd. (Zhongwei, China). The preparation and identification methods are described in the literature (Tian et al. 1995; Huang et al. 1998). First, *Lycium barbarum* polysaccharide (LBP) was separated and refined from *Lycium barbarum* by biochemical methods. Then, five glycoconjugate components (LBP1–LBP5) were separated from LBP by ion exchange chromatography. Then, the five glycoconjugates components (LbGp1–LbGp5) were obtained by gel chromatography and ion exchange chromatography. High-performance liquid chromatography (HPLC) and capillary electrophoresis (CE) showed that these glycoconjugates were homogeneous.

HEK-293 cells and human kidney-2 (HK-2) cells were purchased from Procell Life Science & Technology Co., Ltd. (Wuhan, China). An apoptosis detection kit was purchased from Bestbio (Shanghai, China). SIRT1 and TGF-β1 primary antibodies were purchased from Abcam (Cambridge, UK). FoxO3a, LC3, cleaved caspase-3, and p38 MAPK primary antibodies were bought from Cell Signaling Technology (Danvers, USA).

### Apoptosis in HEK-293 and HK-2 cells was assessed by flow cytometry

HEK-293 and HE-2 cells were split into the control group, DEHP group, DEHP + low-dose LbGp (LbGp(L)) group, DEHP + middle-dose LbGp (LbGp(M)) group, DEHP + high-dose LbGp (LbGp(H)) group, and concanavalin A (ConA) group. ConA, which can promote cell proliferation, served as a positive control for LbGp. The concentrations of DEHP (Amara et al. 2019), LbGp, and ConA (Du et al. 2004) were determined based on previous studies and pre-experiments. Cells in each group were treated with dimethyl sulfoxide (DMSO), DMSO + 200 µg/ml DEHP, DMSO + 200 µg/ml DEHP + 300 µg/ml LbGp, DMSO + 200 µg/ml DEHP + 750 µg/ml LbGp, DMSO + 200 µg/ml DEHP + 1500 µg/ml LbGp, or DMSO + 10 µg/ml ConA.

Cells were resuspended in 400 μl annexin V bundled solution, annexin V (5 μl) was appended and hatched at 4 °C for 15 min (min) under dark conditions, and propidium iodide (PI) (5 μl) was added and incubated at 4 °C under dark conditions for 5 min. Apoptosis was detected by a FACSCanto flow cytometer (BD Biosciences, San Jose, USA) at 24 h, 48 h, and 72 h.

### Preparation of mice

Forty 8-week-old male C57BL/6 J mice (20–22 g) were bought from Zhejiang Vital River Laboratory Animal Technology Co., Ltd. (SCXK (Zhe) 2019–0001) for experiments at the Institute of Laboratory Animal Science, Jinan University (SYXK (Yue) 2017–0174). All mice were maintained at the same temperature (20–24 °C), humidity (40–50%), and 12-h light/12-h dark cycle and were free to eat and drink. After 1 week of accommodative feeding, mice were random split into a control group, DEHP group, DEHP + LbGp group, and flutamide group, with 10 mice in each group. Flutamide has antiandrogenic effects and served as a positive control for DEHP. We determined the doses of DEHP (Jiang et al. 2021), LbGp (You et al. 2019), and flutamide (Reznikov et al. 2011) based on previous studies and pre-experiments. Mice in each group were intragastrically administered peanut oil, peanut oil + 1500 mg/kg DEHP, peanut oil + 1500 mg/kg DEHP, or peanut oil + 50 mg/kg flutamide, respectively, and then subsequently intragastrically administered purified water, purified water, purified water + 100 mg/kg LbGp, or purified water, respectively, 6 h later. The mice were intragastrically gavaged for 14 days. During the experiment, the mice were weighed daily. By the end of the test, mice were anesthetized after fasting overnight, and blood, kidneys, and testicles were collected. Everything possible was done to reduce the pain of the mice. The animal experiment plan was reviewed by the Institutional Animal Care and Use Committee of Jinan University (IACUC-20201215–04).

### Mouse body weight and organ weight

The mice were weighed before death; then, after receiving anesthesia, the mice had their eyes removed and were then bled to death. The kidneys, testicles, and livers were removed and weighed.

### Biochemical tests of the mouse blood

The mice were anesthetized, and their eyes were removed for blood collection. Alanine aminotransferase (ALT), aspartate aminotransferase (AST), albumin (ALB), urea, creatinine (CREA), uric acid (UA), triglyceride (TG), high-density lipoprotein (HDL), low-density lipoprotein (LDL), and glucose (GLU) were determined by an automatic biochemical analyzer. Whole blood specimens were put at room temperature for 2 h and then separated at 4 °C for 855 × G-force (*g*) for 10 min. The supernatant was gathered and put in a fridge at 4 °C for testing. A specific test kit (Rayto Life Sciences Co., Ltd., Shenzhen, China) was used.

### HE staining for analysis of renal and testicular pathology

After the mice were killed, the kidneys and testes were taken out immediately and fixed in 4% paraformaldehyde. After routine dehydration, paraffin was embedded, and 3-μm-thick sections were cut and dyed with hematoxylin and eosin. Pathological variations were watched under a light microscope (Leica Microsystems, Wetzlar, Germany). ImageJ (National Institutes of Health, Bethesda, USA) was utilized to measure the single glomerular cross-sectional area (five fields were randomly selected, the maximum glomerular cross-sectional area was selected, and the mean value was calculated) and the total area of the germ cells (five fields were randomly chosen to calculate the mean total area of the germ cells).

### Masson staining for analysis of collagen deposition in the kidney and testis

The kidneys and testes of mice were fixed with 4% paraformaldehyde, dehydrated, paraffin-embedded, sliced into 3-μm-thick sections, and dyed with hematoxylin, Ponceau, and aniline blue. Collagen deposition was observed under light microscopy. Collagen volume fraction (CVF, the percentage of collagen area to total tissue area) was counted utilizing Image-Pro Plus 6.0 (Media Cybernetics, Maryland, USA).

### IHC staining for assessment of the expression of renal and testicular target proteins

Paraffin sections were dewaxed with xylene and ethanol, and antigens were repaired with citrate buffer and blocked with goat serum. Primary antibodies against SIRT1 (1:500), FoxO3a (1:1600), LC3 (1:500), cleaved caspase-3 (1:400), p38 MAPK (1:400), and TGF-β1 (1:500) were incubated all night at 4 °C. Whereafter, the sections were hatched with secondary antibodies, followed by diaminobenzidine (DAB) staining and restaining with hematoxylin. The sections were then watched under a light microscope. The average optical density (AOD, the integral optical density (IOD) of the yellow portion divided by the total tissue area) was calculated utilizing Image-Pro Plus 6.0.

### Statistical analysis

Data were studied using GraphPad Prism 8 (San Diego, USA). Categorical variables were recorded as percentages and studied by the chi-square test. Continuous variables were recorded as the mean ± standard deviation (SD) and studied by analysis of variance (ANOVA). A *P* < 0.05 was considered statistically significant.

## Results

### LbGp improved DEHP-induced apoptosis of HEK-293 and HK-2 cells

**Fig. 1:**
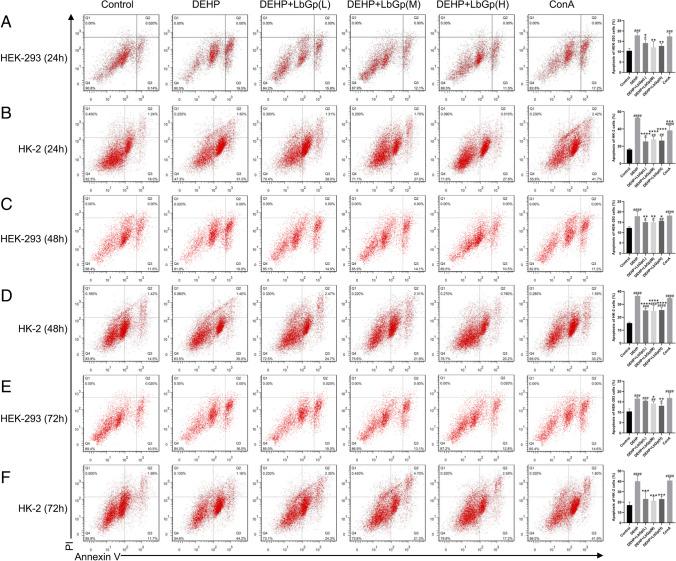
Effect of LbGp on DEHP caused apoptosis in HEK-293 cells and HK-2 cells. The percentage of apoptotic cells treated with DEHP and LbGp at 24 h (**A** and **B**), 48 h (**C** and **D**), and 72 h (E and F) was determined by flow cytometry. ^#^*P* < 0.05, ^##^*P* < 0.01, ^###^*P* < 0.001, and ^####^*P* < 0.0001 in comparison with the control group. ^*^P < 0.05, ^**^P < 0.01, ^***^P < 0.001, and ^****^P < 0.0001 in comparison with the DEHP group. HEK-293, human embryonic kidney-293 cells; HK-2, human kidney-2 cells; DEHP, di(2-ethylhexyl) phthalate; LbGp, *Lycium barbarum* glycopeptide; ConA, concanavalin A; L, low-dose (300 µg/ml); M, middle-dose (750 µg/ml); H, high-dose (1500 µg/ml); PI, propidium iodide

Compared with the control group, HEK-293 and HK-2 cells treated with DEHP at 24 h (*P* < 0.001) (Fig. 1A and B), 48 h (*P* < 0.0001) (Fig. 1C and D), and 72 h (*P* < 0.001) (Fig. 1E and F) showed a significant induction of apoptosis, while LbGp significantly improved the rate of apoptosis in these two cell types at 24 h (*P* < 0.05) (Fig. 1A and B), 48 h (*P* < 0.05) (Fig. 1C and D), and 72 h (*P* < 0.05) (Fig. 1E and F), with no significant difference between the low, middle, or high LbGp doses (*P* > 0.05). ConA significantly increased apoptosis in HEK-293 and HK-2 cells at 24 h (*P* < 0.001) (Fig. 1A and B), 48 h (*P* < 0.0001) (Fig. 1C and D), and 72 h (*P* < 0.0001) (Fig. 1E and F) in comparison with that in the control group.

### Effects of DEHP and LbGp on body weight, kidney weight, testis weight, and liver weight in mice

**Fig. 2:**
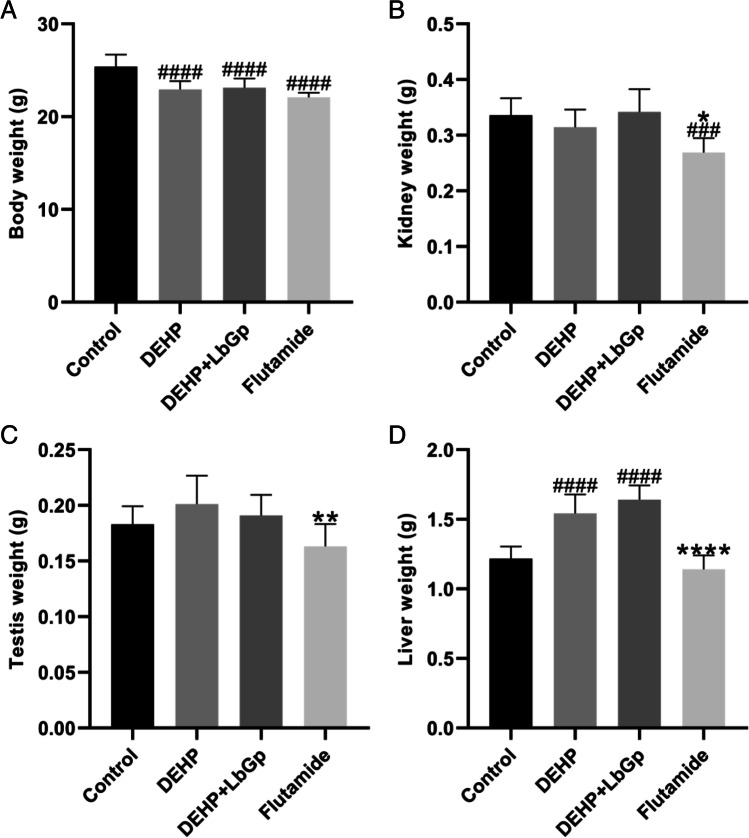
The body weight (**A**), kidney weight (**B**), testis weight (**C**), and liver weight (**D**) of each group at the end point. ^###^*P* < 0.001 and ^####^*P* < 0.0001 in comparison with the control group. ^*^P < 0.05, ^**^P < 0.01, and ^****^P < 0.0001 in comparison with the DEHP group. DEHP, di(2-ethylhexyl) phthalate; LbGp, *Lycium barbarum* glycopeptide

After 14 days of continuous gavage, the body weights of the DEHP group and DEHP + LbGp group decreased by 2.46 g and 2.27 g (*P* < 0.0001), respectively, in comparison to the control group. LbGp improved the body weight but not significantly (Table 1 and Fig. 2A). In comparison to the control group, the liver weight of the DEHP group and the DEHP + LbGp group increased by 0.32 g and 0.42 g (*P* < 0.0001), respectively, and there was no striking variation between the DEHP group and the DEHP + LbGp group (Table 1 and Fig. 2D). DEHP and LbGp had no significant effect on renal weight or testicular weight (Table 1, Fig. 2B and C). Flutamide significantly reduced body weight and kidney weight in comparison to the control group (Table 1, Fig. 2A and B).

**Table 1:** The body weight, kidney weight, testis weight, and liver weight of each group at the end point

Parameter	Control	DEHP	DEHP + LbGp	Flutamide
Body weight (g)	25.39 ± 1.30	22.93 ± 0.91^####^	23.12 ± 1.00^####^	22.09 ± 0.51^####^
Kidney weight (g)	0.34 ± 0.03	0.31 ± 0.03	0.34 ± 0.04	0.27 ± 0.03^###*^
Testis weight (g)	0.18 ± 0.02	0.20 ± 0.03	0.19 ± 0.02	0.16 ± 0.02^**^
Liver weight (g)	1.22 ± 0.09	1.54 ± 0.14^####^	1.64 ± 0.10^####^	1.14 ± 0.10^****^

Data are expressed as the mean ± standard deviation

DEHP, di(2-ethylhexyl) phthalate; LbGp, *Lycium barbarum* glycopeptide

^###^*P* < 0.001 and ^####^*P* < 0.0001 compared with the control group. ^*^P < 0.05, ^**^P < 0.01, and ^****^P < 0.0001 compared with the DEHP group

### Effects of DEHP and LbGp on liver function, renal function, blood glucose, and blood lipids in mice

**Fig. 3:**
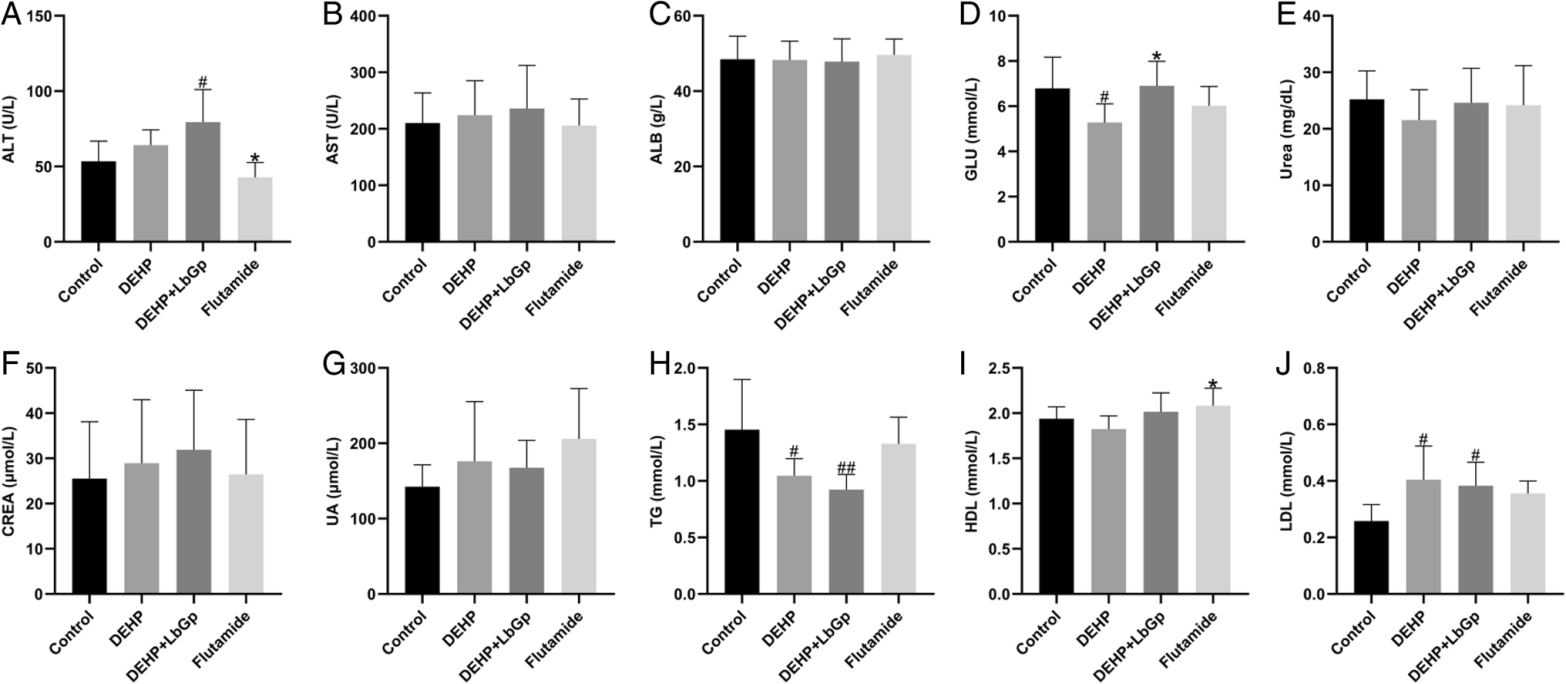
Liver function (**A**–**C**), renal function (**E**–**G**), blood glucose (**D**), and blood lipids (**H**–**J**) in each group at the end point. ^#^*P* < 0.05 and ^##^*P* < 0.01 in comparison with the control group. ^*^P < 0.05 in comparison with the DEHP group. DEHP, di(2-ethylhexyl) phthalate; LbGp, *Lycium barbarum* glycopeptide; ALT, alanine aminotransferase; AST, aspartate aminotransferase; ALB, albumin; GLU, glucose; CREA, creatinine; UA, uric acid; TG, triglyceride; HDL, high-density lipoprotein; LDL, low-density lipoprotein

After 14 days of continuous gavage, ALT in the DEHP group and DEHP + LbGp group increased by 10.8 U/L (*P* > 0.05) and 26.05 U/L (*P* < 0.05), respectively (Table 2 and Fig. 3A). In comparison to the control group, GLU in the DEHP group decreased by 1.5 mmol/L (*P* < 0.05), and in comparison to the DEHP group, GLU in the DEHP + LbGp group increased by 1.62 mmol/L (*P* < 0.05) (Table 2 and Fig. 3D). In comparison to the control group, TG in the DEHP group and DEHP + LbGp group decreased by 0.4 mmol/L (*P* < 0.05) and 0.53 mmol/L (*P* < 0.01), respectively (Table 2 and Fig. 3H). In comparison with the control group, LDL in the DEHP group and DEHP + LbGp group increased by 0.14 mmol/L (*P* < 0.05) and 0.12 mmol/L (*P* < 0.05), respectively, and LbGp improved LDL but not significantly (Table 2 and Fig. 3J). DEHP and LbGp had no significant effect on AST, ALB, urea, CREA, UA, or HDL (Table 2, Fig. 3B, C, E–G, and I). Compared with the control group, flutamide had no significant effect on liver function, kidney function, blood glucose, or blood lipids (Table 2 and Fig. 3A–J).

**Table 2:** Liver function, renal function, blood glucose, and blood lipids in each group at the end point

Parameter	Control	DEHP	DEHP + LbGp	Flutamide
ALT (U/L)	53.38 ± 12.44	64.18 ± 9.46	79.43 ± 19.75^#^	42.79 ± 9.14^*^
AST (U/L)	210.09 ± 49.48	224.06 ± 57.16	235.79 ± 70.76	205.73 ± 43.47
ALB (g/L)	48.46 ± 5.66	48.29 ± 4.64	47.80 ± 5.68	49.61 ± 3.88
GLU (mmol/L)	6.78 ± 1.28	5.28 ± 0.78^#^	6.90 ± 1.01^*^	6.02 ± 0.79
Urea (mg/dL)	25.21 ± 4.66	21.54 ± 5.04	24.61 ± 5.73	24.17 ± 6.51
CREA (μmol/L)	25.51 ± 11.65	28.93 ± 13.13	31.91 ± 12.32	26.43 ± 11.28
UA (μmol/L)	142.45 ± 26.37	176.02 ± 74.22	167.67 ± 33.62	205.95 ± 60.86
TG (mmol/L)	1.45 ± 0.41	1.05 ± 0.14^#^	0.92 ± 0.13^##^	1.33 ± 0.22
HDL (mmol/L)	1.94 ± 0.12	1.82 ± 0.14	2.02 ± 0.19	2.08 ± 0.18^*^
LDL (mmol/L)	0.26 ± 0.05	0.40 ± 0.11^#^	0.38 ± 0.08^#^	0.36 ± 0.04

Data are expressed as the mean ± standard deviation

DEHP, di(2-ethylhexyl) phthalate; LbGp, *Lycium barbarum* glycopeptide; ALT, alanine aminotransferase; AST, aspartate aminotransferase; ALB, albumin; GLU, glucose; CREA, creatinine; UA, uric acid; TG, triglyceride; HDL, high-density lipoprotein; LDL, low-density lipoprotein

^#^*P* < 0.05 and ^##^*P* < 0.01 compared with the control group. ^*^P < 0.05 compared with the DEHP group

#### LbGp improved DEHP-induced renal and testicular pathological damage in mice

**Fig. 4:**
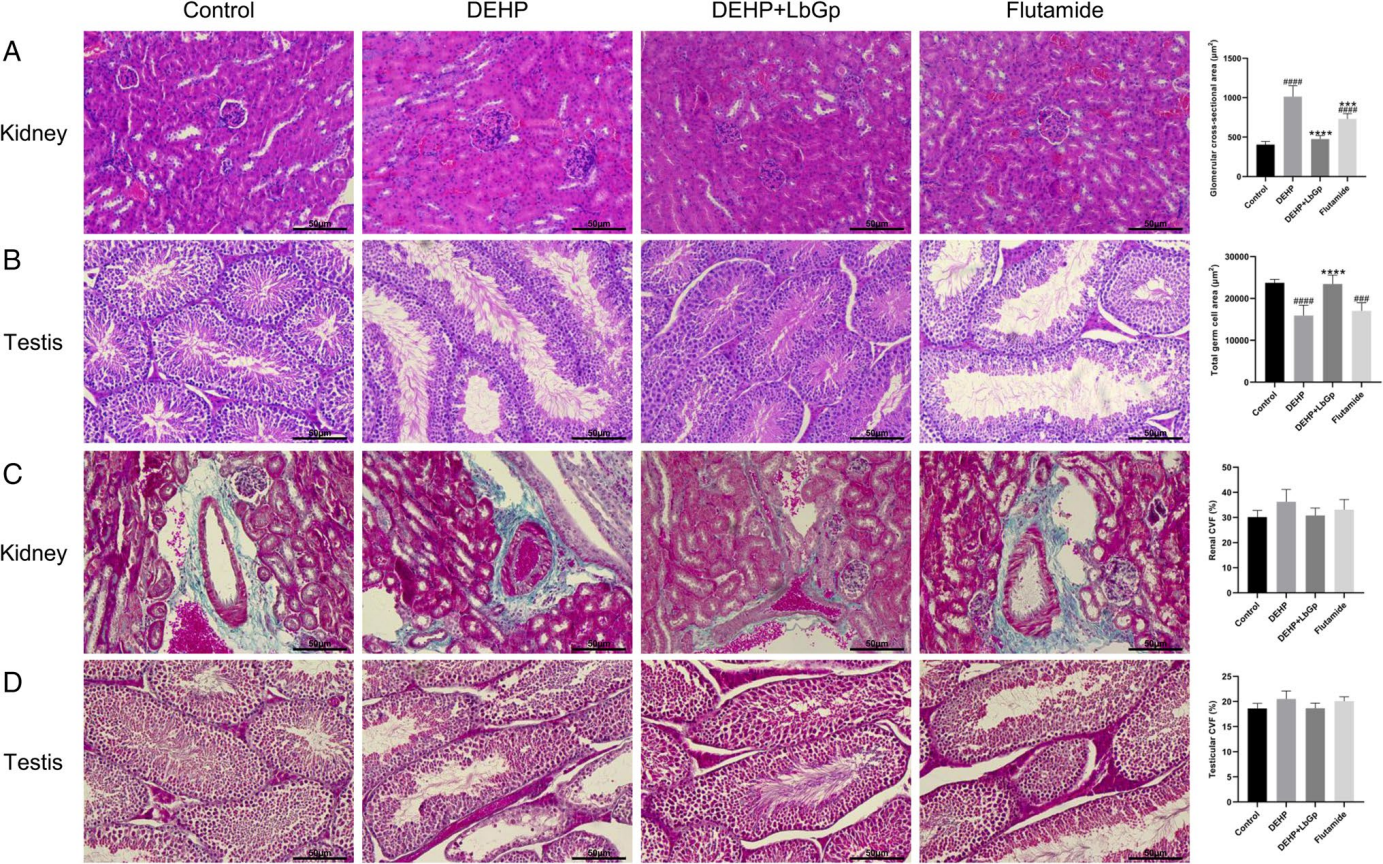
Effect of LbGp on DEHP-induced renal and testicular pathological injury. HE staining of kidney (**A**) and testis (**B**). Masson staining of kidney (**C**) and testis (**D**). Magnification 200 × . The scale is 50 μm. ^###^*P* < 0.001 and ^####^*P* < 0.0001 in comparison with the control group. ^***^P < 0.001 and ^****^P < 0.0001 in comparison with the DEHP group. DEHP, di(2-ethylhexyl) phthalate; LbGp, *Lycium barbarum* glycopeptide; CVF, collagen volume fraction

Hematoxylin–eosin (HE) staining revealed that in the DEHP group, the glomerulus was enlarged, erythrocyte infiltration was increased, dilation from hyperemia was observed, and the renal capsule was dilated, but LbGp improved the renal injury (Fig. 4A). Compared with the control group, the glomerular cross-sectional area in the DEHP group and flutamide group increased by 607.3 μm^2^ (*P* < 0.0001) and 324.3 μm^2^ (*P* < 0.0001), respectively, and decreased by 537.7 μm^2^ (*P* < 0.0001) in the DEHP + LbGp group in comparison with the DEHP group (Fig. 4A). In the DEHP group, the lumen of testicular seminiferous tubules increased, and the germ cells decreased, but LbGp improved testicular injury (Fig. 4B). The total germ cell area in the DEHP group and flutamide group was 7831 μm^2^ (*P* < 0.0001) and 6710 μm^2^ (*P* < 0.001) smaller than that in the control group, respectively, and the total germ cell area in the DEHP + LbGp group was 7520 μm^2^ (*P* < 0.0001) larger than that in the DEHP group (Fig. 4B).

### Effects of DEHP and LbGp on renal and testicular fibrosis in mice

Masson staining revealed that the DEHP group had more collagen fiber deposition in the renal interstitium, while LbGp reduced collagen fiber deposition (Fig. 4C). In comparison to the control group, CVF in the DEHP group and flutamide group raised by 6.04% (*P* > 0.05) and 2.92% (*P* > 0.05), respectively, and decreased by 5.46% (*P* > 0.05) in the DEHP + LbGp group in comparison to the DEHP group (Fig. 4C). Collagen fiber deposition was observed in the testicular interstitium of the DEHP group, and LbGp reduced collagen fiber deposition (Fig. 4D). In comparison to the control group, CVF in the DEHP group and flutamide group raised by 1.90% (*P* > 0.05) and 1.46% (*P* > 0.05), respectively, and decreased by 1.86% (*P* > 0.05) in the DEHP + LbGp group in comparison to the DEHP group (Fig. 4D).

### Effects of DEHP and LbGp on the expression of target proteins in kidney of mice

**Fig. 5:**
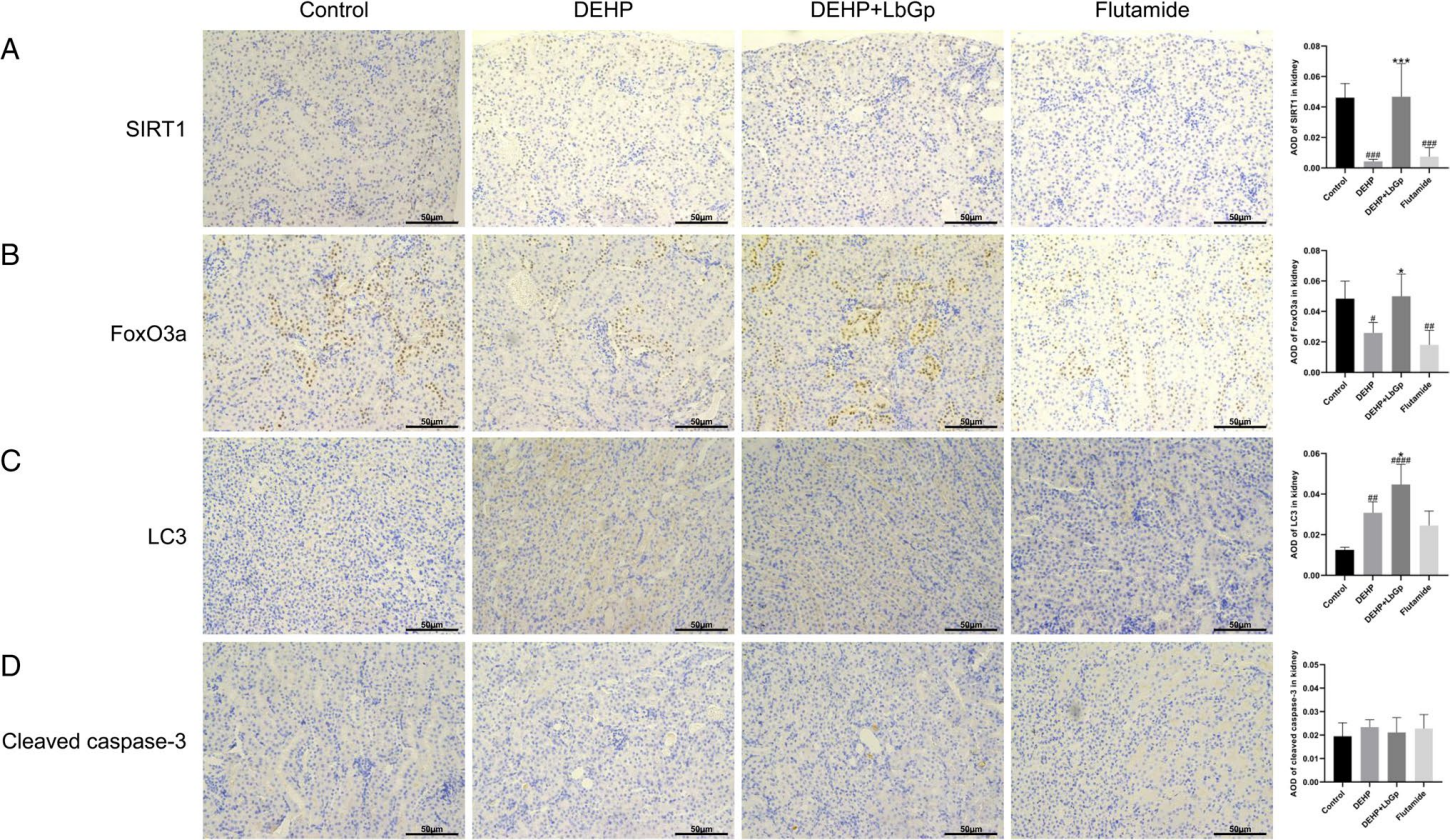
Effects of DEHP and LbGp on the expression of renal autophagy and apoptosis proteins. Immunohistochemical staining results of SIRT1 (A), FoxO3a (B), LC3 (C), and cleaved caspase-3 (D). Magnification 200 × . The scale is 50 μm. ^#^*P* < 0.05, ^##^*P* < 0.01, ^###^*P* < 0.001, and ^####^*P* < 0.0001 in comparison with the control group. ^*^P < 0.05 and ^***^P < 0.001 in comparison with the DEHP group. DEHP, di(2-ethylhexyl) phthalate; LbGp, *Lycium barbarum* glycopeptide; AOD, average optical density

**Fig. 6:**
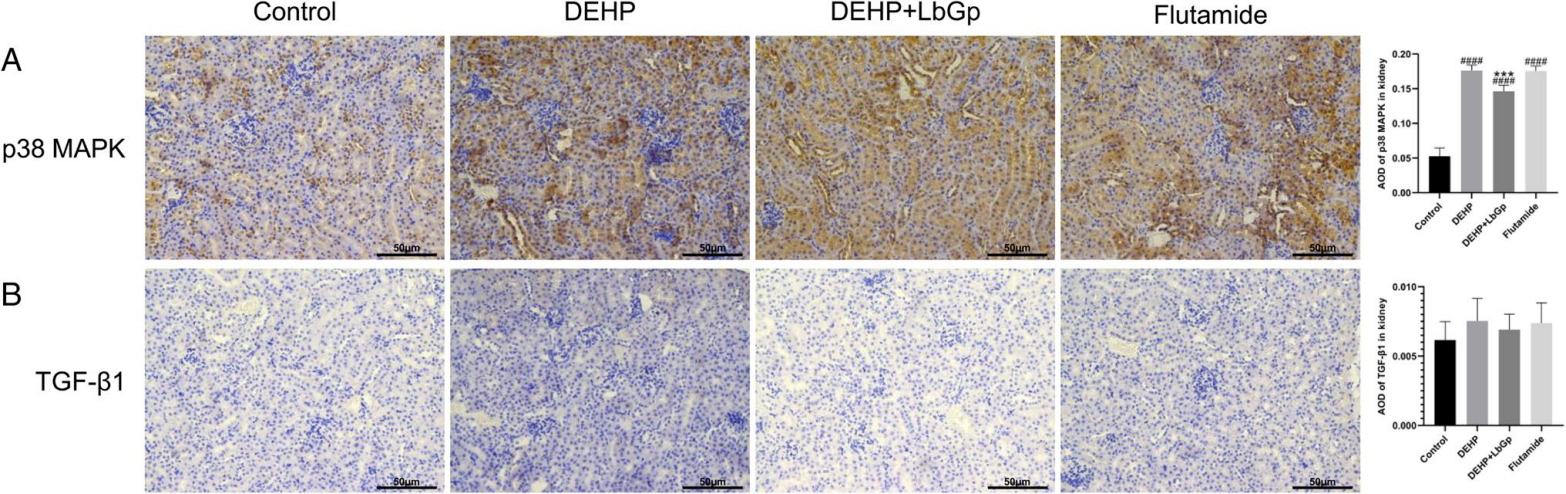
Effects of DEHP and LbGp on renal inflammation and fibrosis protein expression. Immunohistochemical staining results of p38 MAPK (**A**) and TGF-β1 (**B**). Magnification 200 × . The scale is 50 μm. ^####^*P* < 0.0001 in comparision with the control group. ^***^P < 0.001 in comparison with the DEHP group. DEHP, di(2-ethylhexyl) phthalate; LbGp, *Lycium barbarum* glycopeptide; AOD, average optical density

In the immunohistochemistry (IHC) staining, the yellow color represented expression of the target protein. In the kidney, SIRT1 protein expression in the DEHP group and flutamide group decreased by 90.6% (*P* < 0.001) and 83.9% (*P* < 0.001) in comparison to the control group, respectively, and the expression of SIRT1 protein in the DEHP + LbGp group raised by 982.9% (*P* < 0.001) in comparison with the DEHP group (Fig. 5A). FoxO3a protein expression in the DEHP group and flutamide group reduced by 46.3% (*P* < 0.05) and 62.7% (*P* < 0.01) in comparison to the control group, respectively, and increased by 92.5% (*P* < 0.05) in the DEHP + LbGp group in comparison to the DEHP group (Fig. 5B). The expression of LC3 protein in the DEHP group and flutamide group increased by 144.8% (*P* < 0.01) and 94.9% (*P* > 0.05) in comparison to the control group, respectively, and the expression of LC3 protein in the DEHP + LbGp group increased by 45.1% (*P* < 0.05) in comparison with the DEHP group (Fig. 5C). The expression of p38 MAPK protein in the DEHP group and flutamide group was 234.9% (*P* < 0.0001) and 233.2% (*P* < 0.0001) taller than that in the control group, respectively, and the expression of p38 MAPK protein in the DEHP + LbGp group was 17.0% (*P* < 0.001) less than that in the DEHP group (Fig. 6A). Cleaved caspase-3 protein expression and TGF-β1 protein expression were not significantly different between the groups (Figs. 5D and 6B). Compared with the control group, DEHP reduced SIRT1 and FoxO3a expression and increased autophagy factor (LC3) and inflammatory factor (p38 MAPK) expression. In comparison with the DEHP group, LbGp increased the expression of SIRT1 and FoxO3a, further raised the expression of autophagy factor (LC3), and reduced the expression of inflammatory factor (p38 MAPK).

### Effects of DEHP and LbGp on the expression of target proteins in testis of mice

**Fig. 7:**
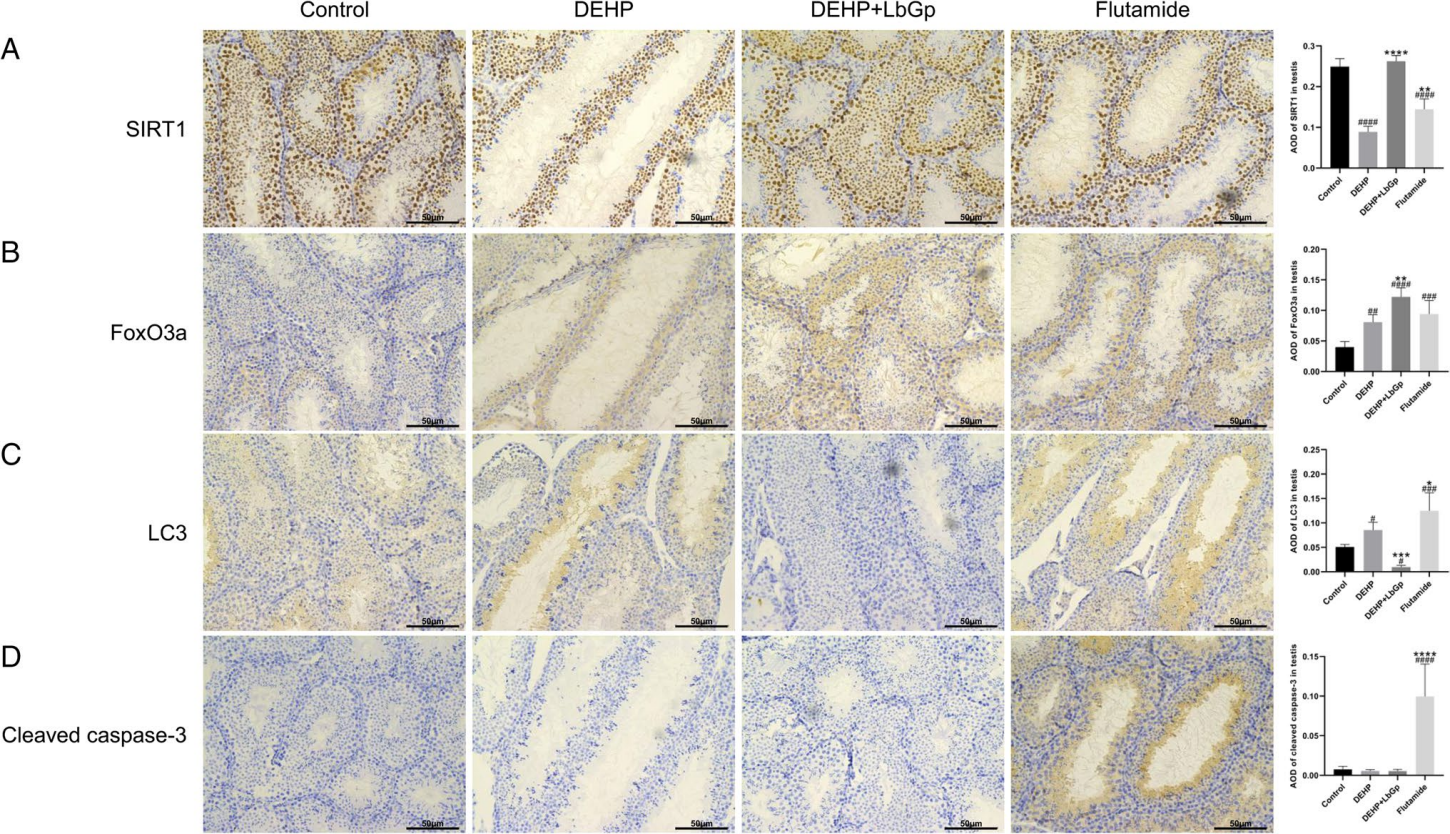
Effects of DEHP and LbGp on autophagy and apoptosis protein expression in the testis. Immunohistochemical staining results of SIRT1 (A), FoxO3a (B), LC3 (C), and cleaved caspase-3 (D). Magnification 200 × . The scale is 50 μm. ^#^*P* < 0.05, ^##^*P* < 0.01, ^###^*P* < 0.001, and ^####^*P* < 0.0001 in comparison with the control group. ^*^P < 0.05, ^**^P < 0.01, ^***^P < 0.001, and ^****^P < 0.0001 in comparison with the DEHP group. DEHP, di(2-ethylhexyl) phthalate. LbGp, *Lycium barbarum* glycopeptide; AOD, average optical density

In the testis, SIRT1 protein expression in the DEHP and flutamide groups decreased by 64.3% (*P* < 0.0001) and 42.1% (*P* < 0.0001) in comparison to the control group, respectively, and increased by 194.4% (*P* < 0.0001) in the DEHP + LbGp group in comparison with the DEHP group (Fig. 7A). In comparison to the control group, FoxO3a protein expression was increased by 102.5% (*P* < 0.01) and 135.9% (*P* < 0.001) in the DEHP and flutamide groups and 51.3% (*P* < 0.01) in the DEHP + LbGp group in comparison to the DEHP group (Fig. 7B). LC3 protein expression in the DEHP group and flutamide group increased by 68.8% (*P* < 0.05) and 146.2% (*P* < 0.001) in comparison to the control group, respectively, and decreased by 88.6% (*P* < 0.001) in the DEHP + LbGp group in comparison with the DEHP group (Fig. 7C). Cleaved caspase-3 protein expression was increased by 1248.8% (*P* < 0.0001) in the flutamide group in comparison to the control group, and there was no striking variation between the control group, DEHP group, and DEHP + LbGp group (*P* > 0.05) (Fig. 7D). In comparison to the control group, DEHP reduced SIRT1 expression and increased FoxO3a and autophagy factor (LC3) expression. Compared with the DEHP group, LbGp increased SIRT1 expression, further increased FoxO3a expression, and reduced the expression of autophagy factor (LC3).

### Discussion

Our study found that DEHP damages the kidney by enhancing p38 MAPK-mediated inflammation, and feedback induces enhanced autophagy and induces excessive autophagy through the SIRT1/FoxO3a pathway to damage the testis. We also found that LbGp protects the kidney by reducing p38 MAPK-mediated inflammation and appropriately enhancing autophagy and protects the testis by regulating the SIRT1/FoxO3a pathway to reduce excessive autophagy. However, the regulation of apoptosis does not seem to be the main mechanism of LbGp reversing DEHP-induced injury. This study reflected the mechanism and possible treatment of DEHP-induced environmental stress on kidney and testis. To our knowledge, no studies have shown the effects and mechanisms of LbGp on renal and testicular injury induced by DEHP.

In this research, we detected that DEHP caused apoptosis in HEK-293 and HK-2 cells at 24 h, 48 h, and 72 h. A previous study found that management of HEK-293 cells with DEHP for 24 h can induce apoptosis by oxidative stress (Amara et al. 2019), which is consistent with this study. In addition, different concentrations of LbGp improved DEHP-induced apoptosis. We did not find any difference in the effects of different doses of LbGp, and the dose–effect relationship needs to be explored further.

Our study found that intragastric administration of 1500 mg/kg/day DEHP for 14 days significantly reduced body weight and increased liver weight in mice but had no significant effect on kidney weight and testicular weight, while LbGp improved body weight loss and further increased the liver weight. Zhu et al. found that intragastric administration of 500 mg/kg/days DEHP for 35 days had no significant influence on the body weight of mice but significantly reduced the weight of the testis and epididymis, which is related to endoplasmic reticulum stress-mediated apoptosis (Zhu et al. 2021). Our study used a higher dose of DEHP, reflecting the body’s stress response to the acute toxicity of DEHP. In addition, LbGp may aggravate the liver injury caused by DEHP, which has not been reported previously.

Our study showed that the ALT level in mice was increased by gavage with 1500 mg/kg DEHP for 14 days. Amara et al. found that intraperitoneal injection of 200 mg/kg DEHP for 30 days significantly increased serum CREA, urea, and LDH levels in mice, and these changes were associated with oxidative stress (Amara et al. 2020). LDH and ALT are indicators of liver damage, suggesting that these results are consistent. However, LbGp further increased ALT, suggesting that DEHP combined with LbGp may cause hepatotoxicity. DEHP significantly reduced GLU and TG in mice. The reason for this may be that DEHP leads to a reduced diet in mice, resulting in lower body weight, GLU, and TG. These results suggest that high doses of DEHP can induce multi-organ stress. In addition, LbGp ameliorated the DEHP-induced GLU decline and LDL increase in mice and further reduced TG. It is suggested that LbGp can restore normal blood glucose and reduce harmful lipid components.

Our study found that 14 days after DEHP gavage, the glomeruli of the mice increased, erythrocyte infiltration increased, dilation from hyperemia was observed, and the renal capsule was dilated, suggesting the presence of glomerular inflammation. These results indicate that DEHP can induce renal inflammatory stress. Jiang et al. found that DEHP intragastric administration for 28 days led to glomerular atrophy and renal tubule dilation in mice (Jiang et al. 2021). Glomerular enlargement is indicative of acute kidney disease, while glomerular atrophy is symptomatic of chronic kidney disease. Different durations of intervention may result in different results. LbGp improved glomerular dilation and renal interstitial fibrosis induced by DEHP, suggesting that LbGp can reduce renal inflammation.

Our study showed that intragastric administration of DEHP for 14 days increased the lumen of seminiferous tubules, decreased the germ cells, and increased collagen fiber deposition in the testes of mice. Zhao et al. found that 14 days of DEHP intragastric administration significantly reduced the diameter of the seminiferous tubule and germ cell layer in rats, which was related to oxidative stress imbalance (Zhao et al. 2020). This is consistent with our study. These characteristics suggest that DEHP-induced stress leads to apoptosis of germ cells. LbGp reduced the enlargement of the seminiferous tubule lumen, increased the number of germ cells, and reduced fiber deposition. These results suggest that LbGp may improve germ cell apoptosis.

Our study found that after 14 days of DEHP treatment, the expression of the upstream regulatory proteins SIRT1 and FoxO3a decreased, and the expression of the autophagy label protein LC3 and inflammation marker protein p38 MAPK increased. However, cleaved caspase-3 and TGF-β1 were not significantly changed. It is suggested that DEHP inhibited the SIRT1/FoxO3a pathway, enhanced renal autophagy and inflammation but had no obvious effect on renal apoptosis and fibrosis. The changes in inflammatory factor p38 MAPK were consistent with the pathological characteristics of inflammation. These results suggested that DEHP induces renal stress by regulating autophagy and inflammation. Amara et al. found that after DEHP treatment, the expression of the apoptosis marker p53 and the proapoptotic protein Bax increased in the mouse kidney, while the expression of the antiapoptotic protein Bcl-2 reduced (Amara et al. 2020). The results showed the same trend as ours. Gu et al. detected that the expression of the inflammatory factors TNF-α and IL-6 was increased in the kidneys of DEHP-treated mice (Gu et al. 2021). It is suggested that DEHP induces renal inflammation, which is consistent with our results. LbGp treatment increased SIRT1, FoxO3a, and LC3 expression, decreased p38 MAPK expression, and had no significant effect on cleaved caspase-3 and TGF-β1 expression. These results suggested that LbGp can activate the SIRT1/FoxO3a pathway to reduce renal inflammation, further enhance renal autophagy, and improve the renal toxicity of DEHP. DEHP-induced renal inflammation can enhance the feedback of autophagy, but not enough to improve renal injury, while LbGp can further enhance autophagy and reduce renal inflammatory injury.

Our study indicated that DEHP significantly reduced SIRT1 expression and increased FoxO3a and LC3 expression in mouse testes but had no significant effect on cleaved caspase-3 expression. These results suggested that DEHP induces testicular stress by regulating autophagy. Zhu et al. found that DEHP could notably increase the apoptosis of testicular germ cells in mice. Cleaved caspase-3 and Bax/Bcl-2 were significantly raised, and Bcl-2 was decreased only at high doses of DEHP, but Bax was not significantly different (Zhu et al. 2021). Balci et al. found that DEHP significantly increased the apoptosis of testicular spermatogenic cells in the progeny of rats and raised the levels of caspase-8, LC3, and Beclin, while there was no difference in the levels of caspase-3 and p62 (Balci et al. 2020). Zhao et al. found that DEHP could notably increase the number of apoptotic cells, increase cleaved caspase-3 levels, and reduce the Bcl-2/Bax proportion in rat testes (Zhao et al. 2020). Zhang et al. found that the quantity of apoptotic cells in the offspring testis of DEHP-treated mice was significantly reduced, while the ratio of Bcl-2 to Bax was not different (Zhang et al. 2020). Fu et al. found that low-dose DEHP upregulated the expression of Beclin-1, Atg7, pULK1, and LC3-II of testicular autophagy proteins in rats. However, a high dose of DEHP has been shown to upregulate the expression of the proapoptotic proteins Bax, cytochrome C, cleaved caspase-9, cleaved caspase-3, and p53 and downregulate the expression of the antiapoptotic protein Bcl-2 (Fu et al. 2020). Due to some differences in these results, the mechanism of testicular autophagy and apoptosis caused by DEHP needs to be further studied. LbGp significantly improved SIRT1 expression, increased FoxO3a expression, and decreased LC3 expression but had no significant effect on cleaved caspase-3 expression. This indicated that DEHP induces excessive autophagy by downregulating SIRT1 and upregulating FoxO3a, leading to testicular germ cell death, while LbGp can restore normal autophagy and protect testicular germ cells by upregulating SIRT1 and FoxO3a.

**Fig. 8:**
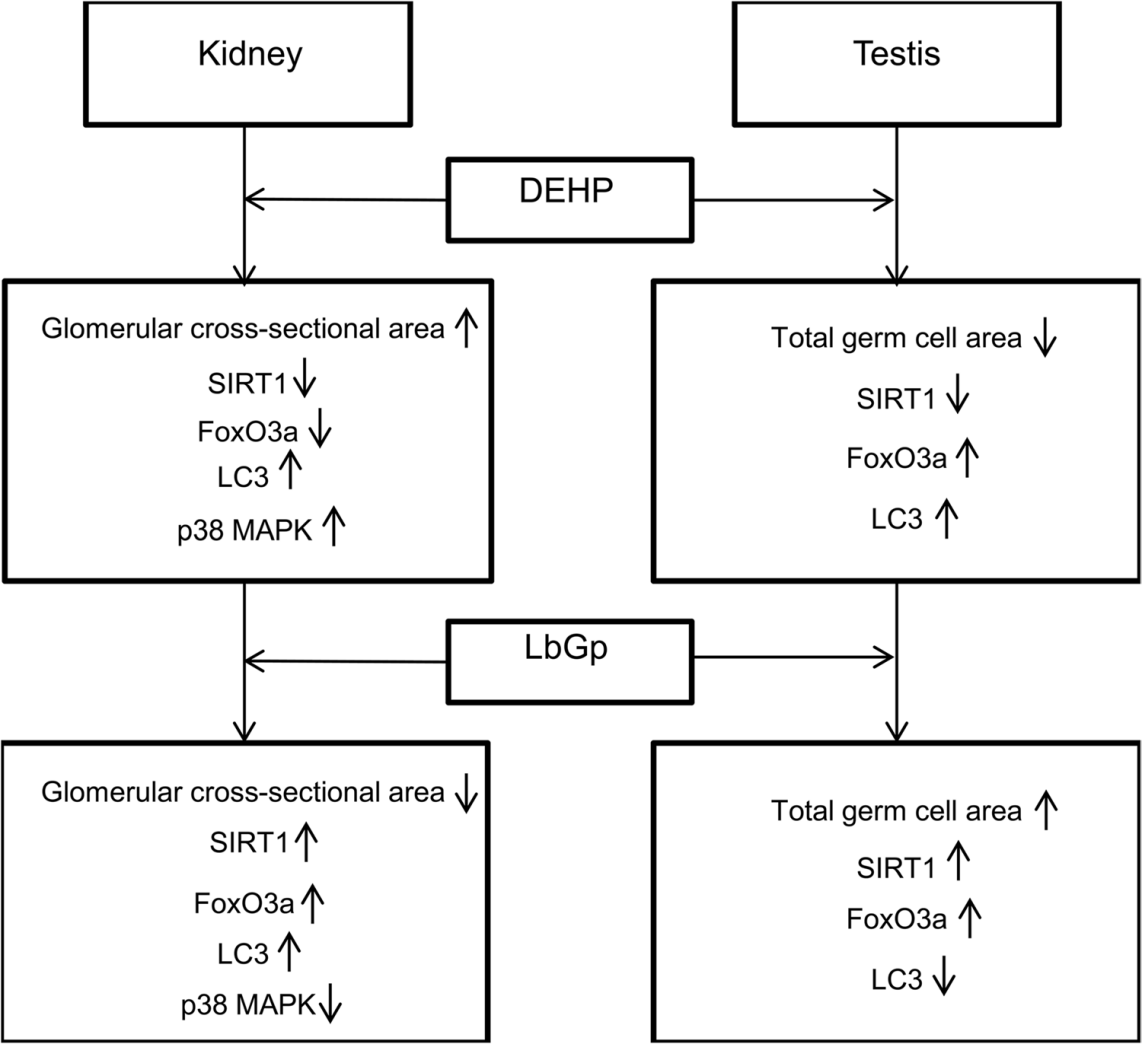
Effect and mechanism of DEHP and LbGp on the kidney and testis. LbGp can ameliorate renal injury induced by DEHP through the p38 MAPK-mediated inflammatory pathway and testicular injury induced by DEHP through the SIRT1/FoxO3a-mediated autophagy pathway. DEHP, di(2-ethylhexyl) phthalate; LbGp, *Lycium barbarum* glycopeptide

In general, DEHP may induce inflammation through the p38 MAPK pathway to damage the kidney and excessive autophagy through the SIRT1/FoxO3a pathway to damage the testis. LbGp ameliorates DEHP-induced renal or testicular injury by modulating the p38 MAPK or SIRT1/FoxO3a pathway (Fig. 8). The mechanisms of DEHP and LbGp regulating renal and testicular autophagy and apoptosis need to be further investigated.

There are some shortcomings in this study. First, the mechanism of action was not studied in vitro. Second, in vivo gene knockdown or knockout studies were not conducted. Third, no high-throughput sequencing was performed to identify candidate genes. Since this study focused on in vivo experiments, we did not study the mechanism of action in in vitro experiments. In vivo experiments involving high-throughput sequencing and gene knockdown will be carried out in follow-up studies by our research group.

## Conclusion

In conclusion, LbGp can significantly improve kidney and testicular injury induced by DEHP. The mechanism of DEHP injury to the kidney and testis is related to the p38 MAPK-mediated inflammatory response and SIRT1/FoxO3a-mediated excessive autophagy, respectively. LbGp protects the kidney by reducing inflammation and appropriately enhancing autophagy and the testis by reducing excessive autophagy.

## Data Availability

The data that support the findings of this study are available from the corresponding author upon reasonable request.
